# Social modulation of decision-making: a cross-species review

**DOI:** 10.3389/fnhum.2013.00301

**Published:** 2013-06-26

**Authors:** Ruud van den Bos, Jolle W. Jolles, Judith R. Homberg

**Affiliations:** ^1^Department of Organismal Animal Physiology, Faculty of Science, Radboud University NijmegenNijmegen, Netherlands; ^2^Department of Zoology, University of CambridgeCambridge, UK; ^3^Department of Cognitive Neuroscience, Centre for Neuroscience, Donders Institute for Brain, Cognition, and Behaviour, UMC St. RadboudNijmegen, Netherlands

**Keywords:** decision-making, translational research, social environment, stress, psychological, humans, animals

## Abstract

Taking decisions plays a pivotal role in daily life and comprises a complex process of assessing and weighing short-term and long-term costs and benefits of competing actions. Decision-making has been shown to be affected by factors such as sex, age, genotype, and personality. Importantly, also the social environment affects decisions, both via social interactions (e.g., social learning, cooperation and competition) and social stress effects. Although everyone is aware of this social modulating role on daily life decisions, this has thus far only scarcely been investigated in human and animal studies. Furthermore, neuroscientific studies rarely discuss social influence on decision-making from a functional perspective such as done in behavioral ecology studies. Therefore, the first aim of this article is to review the available data of the influence of the social context on decision-making both from a causal and functional perspective, drawing on animal and human studies. Also, there is currently still a gap between decision-making in real life where influences of the social environment are extensive, and decision-making as measured in the laboratory, which is often done without any (deliberate) social influences. However, methods are being developed to bridge this gap. Therefore, the second aim of this review is to discuss these methods and ways in which this gap can be increasingly narrowed. We end this review by formulating future research questions.

## Introduction

Decision-making plays a pivotal role in daily life and comprises a complex process of assessing and weighing short-term and long-term costs and benefits of competing actions. The output of the decision-making process, i.e., which action is to be taken, is determined by an interaction between impulsive or emotionally based systems, responding to immediate (potential) rewards and losses or threats, and reflective or cognitive control systems controlling long-term goals (Bechara, 2005; de Visser et al., 2011). Decision-making is influenced by many factors. However, whereas factors such as sex, age, genotype, and personality have been extensively investigated and discussed (reviews; Crone and van der Molen, 2004; Overman, 2004; Overman et al., 2004; de Visser et al., 2011; Homberg, 2012; van den Bos et al., 2013a), relatively little attention has been paid to the crucial moderating effect of social context on decision-making. This is all the more surprising given that decisions in real life are often strongly influenced by the social environment and involve direct and indirect social interactions.

The social environment may affect decision-making in different ways. For instance, decisions may directly involve social partners such as when deciding to share knowledge or goods with others or to provide support (review; Rilling and Sanfey, 2011). Furthermore, subjects may adjust their decisions depending on who is with them or who they consider as their reference-point at the time of the decision. For instance, in the case of so called “conformity behavior,” subjects change their behavior to match that of the rest of the group (Morgan and Laland, 2012). Finally, the social environment may influence decisions globally by “setting the atmosphere.” For instance, the social environment may breathe a tense or relaxed atmosphere, which influences the individual's emotional state and thereby its decisions (review; Starcke and Brand, 2012). While studies in the field of behavioral ecology have provided elaborate understanding of functional aspects of the social context of decision-making behavior, studies in the field of neuroscience have begun to provide information on the causal aspects and the neural substrate underlying decision-making behavior in a social context. Still, crosstalk between these fields rarely occurs. Researchers in both fields may benefit from insights from both domains that will enable progress toward a common understanding of the social modulating role on decision-making. Therefore, the first aim of our review is to discuss the influence of the social context on decision-making both from a causal and functional perspective, drawing on animal and human studies.

Currently, there is still a gap between decision-making in real life where influences of the social environment are extensive, and decision-making as measured in the laboratory, which is often done without any (deliberate) social influences. Subjects may for instance be less disturbed by stressful conditions when in company of friends or relatives with thereby little effect on their decisions in real life, while showing high levels of stress and concomitant effects on decision-making in the laboratory when tested singly. While these laboratory findings may be important for studying basic mechanisms of e.g., the effects of stress on decision-making (Preston et al., 2007; Lighthall et al., 2009; van den Bos et al., 2009), they hamper for instance assessing the value and general applicability of laboratory findings to the functioning of people, such as patients, in daily life. Furthermore, they miss out the important impact the social environment may normally play on individual and group decision-making. However, a major obstacle to assess the role of the social environment in decision-making under laboratory conditions in humans is that it is difficult to create ecologically valid conditions. Therefore, monitoring real life effects of the social environment on decision-making would be a significant step forward. In rodents, home-cage experimental set-ups, which allow for careful manipulation of brain-behavior relationships in social settings, have been developed as means of bridging precisely this gap. Therefore, a second aim of our review is to discuss these developments in methodology to address the question of the effect of the social environment on decision-making.

Given the foregoing, in the following sections we will discuss how the social environment may modulate decision-making and how this can be incorporated in experimental studies. In section Decision-making in a social context, we will discuss direct and indirect social influences on decision-making, while in section Social stress and decision-making the effects of social stress on decision-making are addressed. Where possible we link a causal and functional perspective and discuss underlying neural substrates. In section Laboratory studies and real-life studies we will (briefly) discuss ways to incorporate the social environment into studies of decision-making. We end this review (section Concluding remarks) with a brief summary of the main issues addressed and define (some) future questions.

## Decision-making in a social context

Humans are an exceptionally successful species, both in the number of individuals and in our flexibility to expand to the range of environments and situations in which we live. A major factor underlying this success boils down to our complex social life as we have the ability to acquire valuable knowledge and skills from others through social learning and teaching and build upon this generation after generation (Boyd and Richerson, 1985; Laland et al., 2011). In our daily life we constantly make decisions based on our personal information and experience as well as that of others, i.e., social learning. Our behavior may be restricted through social conformity (Asch, 1956), or promoted or enhanced through facilitation (Zajonc, 1965). Furthermore, often the decisions of multiple individuals may result in collective behavior, such as the synchronization of applause (Néda et al., 2000), or have to be made jointly to reach a consensus (Conradt and Roper, 2005; Dyer et al., 2008). Living with others comes with the potential benefit of cooperation (Fehr and Fischbacher, 2004a) as well as costs of competition when resources are limited (Davies et al., 2012). Finally, an individual's decisions may be indirectly influenced by the social environment, by affecting an individual's emotional state. Importantly, the modulating role of the social environment is strongly affected by an individual's characteristics and personality as well as that of its group mates (Webster and Ward, 2011).

To fully understand the role of social modulation on decision-making, it is important to consider it from both a causal and functional perspective (Tinbergen, 1963; see e.g., Morgan and Laland, 2012). In neuropsychology, functional explanations are rarely taken into account while this behavioral ecological perspective may help to understand how the behavior of individuals is adapted to the social environment in which they live (Davies et al., 2012). A growing list of behaviors once described as uniquely human have now been described in a range of animals, such as teaching (Franks and Richardson, 2006; Thornton and McAuliffe, 2006), culture (see Laland, 2008; Laland et al., 2011), and conformity (Whiten et al., 2005; Galef and Whiskin, 2008; Jolles et al., 2011), which provide us with new insights into our own behavior. Therefore, the next few sections are focused on a behavioral ecological perspective with links to relevant human and animal laboratory studies. However, as the human literature on social decision-making has been reviewed elsewhere, we limit ourselves to the most relevant human experimental studies (see e.g., Fehr and Fischbacher, 2004a,b; Lieberman, 2007; Frith and Singer, 2008; Behrens et al., 2009; Rilling and Sanfey, 2011).

### Social learning

For social species, like humans, the social environment plays a critical role in day-to-day decision-making, such as where to live, what to eat and with whom to mate, and may affect their emotional state (see section Observational fear learning). Decisions can be based on either personal experience and/or information gathered by others (Danchin et al., 2004) and through “social learning,” individuals may for example learn how (observational learning) to deal with a resource or where it is located (local enhancement; Thorpe, 1956; Webster and Laland, 2012). Although social learning may involve several different learning mechanisms (Laland, 2008) only some rely on advanced cognitive abilities (Galef, 1988; Heyes, 1994) and most cases appear to result from very simple processes (Galef, 1988). Indeed, although social learning may seem particular to humans, animals from a broad range of species gather and exploit information generated by others (review; Galef, 1988; Heyes, 1994; Heyes and Galef, 1996).

A considerable part of the social learning literature has been performed with rats (review; Galef and Giraldeau, 2001; Galef, 2007) and has shown that rats use information from others to learn where, what, how and even when to eat (Galef and Giraldeau, 2001). Both the social information provided by visual and olfactory cues from conspecifics provide a strong basis for individual foraging decisions. Just by observing conspecifics, rats quickly locate food and join to feed with them (see Galef and Giraldeau, 2001). This is further intensified by deposited olfactory cues on both the food and the location of the food (Galef, 2007), which may for example enable young rats to learn what foods are best to eat as they may not be able to figure this out by themselves (see Galef, 2007). In particular the olfactory cues via the breath of conspecifics may result in these socially induced food preferences that may overrule personal preferences (Galef and Whiskin, 2008; Jolles et al., 2011) and even reverse learned aversions to foods (Galef, 1986).

To accurately make decisions, individuals need to constantly weigh the costs and benefits of private and social information and need to be selective when and whom to copy (Galef, 1995; Laland, 2004). Social learning may be beneficial as it allows individuals to acquire relevant information without having the risk or costs associated with individual learning. However, social information may be outdated, for example when the environment is highly variable, or less valuable, when the environment is very stable (Boyd and Richerson, 1985). Thus, relative reliance on social and individual learning can be viewed as involving a trade-off between accuracy and cost (Boyd and Richerson, 1985; Laland, 2004; Kendal et al., 2005). For example, Dally and colleagues (2008) showed that rooks selectively consumed the same food as a demonstrator when the foods were novel, but not when the foods were familiar. Likewise, Galef and Whiskin showed that the greater the discrepancy between private and social information, the less likely the subject is to behave in accord with the socially acquired information (Galef and Whiskin, 1998). Moreover, Brown and colleagues (2008) showed that personal and social information about spatial choices are combined in a rat's working memory and both the quality of the food available and the memory of a familiar conspecific's behavior affect an individual's tendency to visit spatial locations in a radial-arm maze.

The trade-off between accuracy and costs is nicely illustrated by the difference in public information use of two closely related species of sticklebacks. Coolen and colleagues (Coolen et al., 2003) showed that while nine-spined sticklebacks exploited public information and foraged at the areas they observed others to have better feeding rates, three-spined sticklebacks ignored this information and relied in their decisions on their own experience. This difference in social information use may be explained by the relative difference in costs of self-acquired information between the two stickleback species. The robust defenses that three-spined but not nine-spined sticklebacks have, such as large spines and armored body plates, allows them to sample alternative food patches directly in relatively better safety, as reflected by the increased time nine-spines spent hidden amongst vegetation (Laland, 2008).

When the presence of group mates affects the behavior of an individual, allowing or causing them to engage in certain behaviors at a different rate, or to perform behaviors that they would not perform at all if they were alone, this is called social facilitation (Zajonc, 1965). For example, in animals it has been shown that the presence of others may result in higher activity (Griffiths and Foster, 1998; Webster et al., 2007), increased foraging (Webster et al., 2007; Dally et al., 2008) and provide scrounging opportunities (review; Giraldeau and Caraco, 2000). For example, conform to human work, studies on rats have shown that the greater the number of models and the greater their uniformity in behavior, the more likely a naive subject will act in accord with the information that conspecifics provide (Galef and Whiskin, 1995). These changes in behavior can probably be ascribed to proximate mechanisms such as greater anti-predator benefits of larger groups (review; Krause and Ruxton, 2002), investment in vigilance and/or increased competition (review; Beauchamp, 2003). This is nicely illustrated by two studies in ravens (Stöwe et al., 2006a,b) which showed that when individuals were alone compared to in a group, they approached a novel object faster but spent less time close to and manipulating it. Although the social group enabled individuals to decrease time investment in vigilance, they may have a higher approach latency because individuals might wait for the other to take the risk and lead.

### Conformity behaviour

Social learning theory suggest that in most circumstances where natural selection favors reliance on social learning, conformity is favored and individuals, both humans and other animals, should adopt the behavior of the majority (Boyd and Richerson, 1985; Laland, 2004). This particular form of social modulation on decision-making is especially important as it has been argued to be a major mechanism in human cultural evolution (Boyd and Richerson, 1985; Efferson et al., 2008). One of the earliest described studies on human conformity was performed by Asch (1955, 1956). In a very influential paper, Asch (1955) described how adults would be willing to abandon their own perceptual judgment in a simple visual task and go with the overtly false alternative as a result of group normative behavior. Since then a huge number of studies has replicated these kinds of findings (see Bond and Smith, 1996; Morgan and Laland, 2012). Interestingly, the extent of conformity behavior seems to be strongly dependent on the situation. Namely, if a participant has to make a public response and is face-to-face with the majority, there is a strong normative influence of conformity, whereas it is weaker when participants make a private response and indirectly communicate with the majority (Bond, 2005). Furthermore, conformity behavior may be dependent on task difficulty and its importance (Baron, 1996), group size (Asch, 1955; Bond, 2005) and culture (Bond and Smith, 1996) among others.

Recently, several studies have addressed the neurobiological basis of conformity (see also Morgan and Laland, 2012). For instance, studies using mental rotation and auditory tasks (Berns et al., 2005, 2010) showed that social information may affect brain regions classically associated with perception as well as the processing areas associated with each task, suggestion that social information was affecting the subjects' perception as well as decision-making (see Morgan and Laland, 2012). Moreover, it has been shown that while cingulate areas are involved in monitoring the difference between private and public information (Klucharev et al., 2009), the ventral striatum is involved in the tendency to adjust one's behavior to the social information (Burke et al., 2010; Campbell-Meiklejohn et al., 2010), which may be related to rewarding aspects of being in line with the behavior of others (Klucharev et al., 2009; Burke et al., 2010; Campbell-Meiklejohn et al., 2010).

Conformity has been described in a wide range of animal species including fish (Laland and Williams, 1998; Day et al., 2001; Pike and Laland, 2010), rats (Galef and Whiskin, 2008; Jolles et al., 2011) and primates (Whiten et al., 2005; Dindo et al., 2009) (see Webster and Ward, 2011 for a review). For example, Laland and Williams (1997) showed that guppies preferentially chose a foraging route they had previously observed demonstrators use despite an equally valid available alternative. Individuals may base these kind of conformity decisions on heuristic rules of social attraction (Webster and Laland, 2012) such as to approach others (e.g., Laland and Williams, 1997), to approach larger over smaller groups (e.g., Lachlan et al., 1998; Day et al., 2001) and to approach groups that produce cues indicative of higher foraging success (e.g., Coolen et al., 2003, 2005). These tendencies are likely to benefit animals in most cases as it allows them to detect food without having to pay the costs of sampling the environment directly (see e.g., Pitcher et al., 1982; Day et al., 2001). However, sometimes this conforming to the behavior of others may come with “opportunity costs.” For example, individual fish may discover a visually isolated food patch faster and exploit it for longer than when a group of conspecifics is present (Webster and Laland, 2012), and smaller groups may discover a hidden food patch more quickly than larger ones (Day et al., 2001). The reliance on social information may sometimes even result in individuals to base their decisions on maladaptive information, such as rats consuming less palatable and sodium-deficient diets based on the breath of conspecifics (Galef, 1986), and even after the source of information is removed, such as guppies that kept on using energetically costly routes to food patches despite shorter alternatives available (Laland and Williams, 1998).

Although conformity of the basic “follow the majority” kind has been demonstrated in a variety of species of which Pike and Laland's (2010) study on public information use in sticklebacks provides compelling evidence, only a few animal studies (Whiten et al., 2005; Galef and Whiskin, 2008; Jolles et al., 2011) have investigated the situation where conformity overrides the discovery of valid alternative means (cf. Asch, 1955, 1956). In a two-action diffusion study in chimpanzees, Whiten and colleagues (2005) showed that although some individuals discovered an alternative technique to free trapped food items to the one seeded in their group, they later re-converged on the norm of their group, demonstrating conformity in the face of discovering a functional alternative. Two recent studies also suggest the existence of this type of conformity in rats (Galef and Whiskin, 2008; Jolles et al., 2011). Rats were given the opportunity to learn that two diets differed in palatability. They were subsequently exposed to a demonstrator that had eaten the less palatable food and were thereafter exposed to the same diets again. By simply being exposed to the odors in the breath of a conspecific for 30 min, individuals considerably decreased their preference for the more palatable food. Interestingly, despite similar initial preferences and similar social information, some rats were more resistant to changing their preference in relation to private and social information than others (Jolles et al., 2011), suggesting a different sensitivity to conflicting information (cf. Klucharev et al., 2009).

### Collective behaviour and group decision-making

Both humans and many group living animals exhibit complex, coordinated, group patterns, such as lanes of traffic flow in human crowds (Helbing and Molnar, 1995) and the three-dimensional movements of fish shoals (Couzin and Krause, 2003). Through collective action, individuals can enhance their capacity to detect and respond to salient features of the environment, resulting in more accurate decision-making (Couzin, 2009) without the need of explicit signals or complex communication (Couzin et al., 2005; Dyer et al., 2008). The common property of these phenomena is self-organization, suggesting that much of complex group behavior may be coordinated by relatively simple interactions among the members of the group (review; Couzin and Krause, 2003). Indeed, recently studies have begun to reveal that collective decision-making mechanisms across animal species, from insects to birds and even humans, seem to share similar functional characteristics (Couzin and Krause, 2003; Conradt and Roper, 2005; Sumpter, 2006). For example, Helbing and colleagues (Helbing and Molnar, 1995; Helbing et al., 2000) have shown that simple rules such as “try to minimize travel time,” “avoid collisions” and “move in the direction of other people” may help explain pedestrian movements on busy streets and in life-threatening situations. Similar patterns have been described for non-human animals including the spectacular trails of ants on foraging trips (Couzin and Franks, 2003), the collective movements of starlings (Ballerini et al., 2008), and social interactions in shoaling fish (Herbert-Read et al., 2011).

In some cases group decisions are the result of a consensus reached by the individuals in the group (Conradt and Roper, 2005). Humans make these kinds of decisions all the time, from agreements in groups of a few people, to large-scale international conventions and political elections. However, also amongst non-human animals consensus decision-making is very common, such as travel routes in navigating birds use and the timing of activities (review; Conradt and Roper, 2003, 2005). In many situations conflicts may exist between the preferences of different individuals (Couzin et al., 2005). However, all individuals in the group have to decide on the same action because the group will fall apart unless a consensus is reached (Conradt and Roper, 2005), resulting in a loss of many of the advantages of group living (review; Krause and Ruxton, 2002). In line with theoretical predictions (Couzin et al., 2005), it has now been demonstrated that only a small proportion of knowledgeable individuals is needed to influence the direction of movement of the whole group, such as has been shown for nest site choice in social insect colonies (Franks et al., 2003; Seeley, 2003), the foraging movements in golden shiner fish (Reebs, 2000), and humans moving to a target without the use of verbal communications or obvious signaling (Dyer et al., 2008).

### Cooperation and competition

An important way to understand social decision-making in humans and other social animals is to look at it in terms of costs and benefits, not only to the actor as indicated above, but also to the recipient in the social context (Hamilton, 1964; West et al., 2007; Davies et al., 2012). For this it is important to keep in mind that via natural selection those genes are favored that increase an organism's ability to survive and reproduce (fitness). Therefore, individuals will often attempt to act in such a way as to receive immediate, selfish benefits, which may often result in competition or mutualistic cooperation. This is nicely illustrated by the Prisoner's Dilemma (PD; Axelrod and Hamilton, 1981) in which individuals can either cooperate or defect. Both individuals would benefit from mutual cooperation but both are also tempted to cheat, as it would be more rewarding to the individual. Therefore, irrespective of the other player's choice, it pays to defect. This raises the problem why cooperation is so common among human and animal societies (see West et al., 2007) and why individuals not act selfishly all the time and exploit the cooperative behavior of others (see Davies et al., 2012). In many cases, the cooperating individual simply acts selfishly and gains an immediate benefit, but thereby provides by-product benefits to its group mates, such as the benefits of an increased group size, i.e., reduced chance of predation, due to helping behavior in meerkats (Clutton-Brock, 2002). When on the other hand cooperation is altruistic—costly to the cooperator and beneficial to the recipient—cooperating individuals may still gain selfish benefits in the long term by using conditional strategies (Stevens and Hauser, 2004), such as cooperating only with relatives (kin selection; Hamilton, 1964), interacting only with those that have cooperated previously (reciprocity; Trivers, 1971; see Clutton-Brock, 2009), or under enforcement (Frank, 2003).

Individuals may help relatives as this may increase their genetic representation in future generations, and thus their fitness, as relatives share genes by common descent (see further Hamilton, 1964; West et al., 2007; Davies et al., 2012). If individuals preferentially help those that have helped them or those that help others, also known as reciprocity, the short-term cost of being cooperative is outweighed by the long-term benefit of receiving cooperation (Trivers, 1971). Although the PD has shown that when individuals meet only once it is better for individuals to defect than to cooperate, some form of cooperation may be stable if there is a chance both players will meet again because the long-term benefits of cooperation may outweigh the short-term benefit of defecting (Axelrod and Hamilton, 1981). Indeed, experimental work on both humans (Fehr and Fischbacher, 2003) and rodents (Rutte and Taborsky, 2007, 2008; Viana et al., 2010) has shown that individuals cooperate at higher levels in repeated interactions. For example, Rutte and Taborsky showed that rats that were trained to pull a stick in order to produce food for a partner pulled more often for an unknown partner after they were helped than if they had not received help before (generalized reciprocity; Rutte and Taborsky, 2007) and more often from a partner they received help from (direct reciprocity; Rutte and Taborsky, 2008). Furthermore, Schneeberger and colleagues (2012) showed that, similar to human PD studies, rats provided more food to cooperative partners than to defectors and that furthermore, this was dependent on costs: when rats experienced experimentally increased resistance to pull the stick of the apparatus and deliver food to the social partner, they reduced their help. It remains unclear, however, to what extent these behaviors may potentially be ascribed to simpler processes such as conditioned place preference. For example, rats have been shown to prefer a social partner over an empty space (Trezza et al., 2009) and to cooperate 80% of the time if they have the choice to act either alone or in cooperation with a social partner to obtain food pellets (Tsoory et al., 2012). Indeed, although reciprocity has attracted a huge amount of attention, it is thought to be generally unimportant outside humans (Hammerstein, 2003; Stevens and Hauser, 2004) as in most cases cooperation can be explained by more simple mechanisms such as by-product-benefits (Hammerstein, 2003; Clutton-Brock, 2009). Nevertheless, it shows that (lab) rodents may provide a good model system to investigate the mechanisms and development of cooperation (Łopuch and Popik, 2011).

Finally, enforcement or punishment may alter the benefit/cost ratio of helping and thereby favor cooperation (Frank, 2003). The consequences of punishment are nicely illustrated in cleaner fish. Cleaner fish remove parasites on the body of other species of fish that cannot remove the parasites themselves. Although, the cleaner fish prefer to eat parts of their clients' tissue they rarely perform this cheating behavior as their hosts may punish them by chasing them or by swimming away (Bshary and Grutter, 2002). What may be special about human cooperation is that we have the capacity to establish and enforce social norms (Fehr and Fischbacher, 2003, 2004a,b) because our societies are based on large-scale cooperation among genetically unrelated individuals (Henrich et al., 2003). For example, human research investigating the conditional cooperation on social norms has shown that subjects increase their contribution to the public good if the average contribution of the other group members increases (see Fehr and Fischbacher, 2004a). Moreover, third-party punishment experiments in which the PD is extended with a passive third party has shown that these individuals punish not-cooperating players despite a cost to themselves and that moreover, defection was punished much more severely if the other player cooperated than if they both defected (Fehr and Fischbacher, 2004b).

When individuals act selfishly under situations of limited resources, competition may occur between individuals. Competing individuals have to weigh the competitive efforts against expected benefits as well as the intensity of the conflict. Individuals may compete by exploitation and/or by resource defense (Davies et al., 2012). Importantly, the best way for an individual to behave often depends on what its competitors are doing (review; Davies et al., 2012), which will therefore result in a stable outcome of competition, also known as the evolutionary stable strategy (EES; Maynard Smith and Price, 1973). Under ideal free distributions in which individuals are free to go where they want and have complete information about the availability of resources (Fretwell, 1972), individuals will distribute themselves in such a way that all individuals have the same rate of resource acquisition. For example, people queuing at the checkout area of the supermarket will often decide to choose the shorter and faster queues, ultimately resulting in all queues being of more or less equal length. However, in most cases individuals may not be free to go where they want as better competitors will occupy the richer habitats. This situation is very common in the natural world (see Davies et al., 2012). For example, although ducks have been shown to occur in stable distributions of individuals among foraging sites (Harper, 1982), some ducks were better competitors than others and grabbed most of the food (Harper, 1982). Importantly, defense of a resource has costs as well as benefits and individuals should only behave territorial when the benefits are greater than the costs. This may also help explain why often variable competitive behavior can be found within a population, such as producers and scroungers in a foraging context (Giraldeau and Caraco, 2000), as the costs and benefits may be different between individuals.

Insight in the neural mechanisms underlying cooperation and competition is increasing (see Rilling and Sanfey, 2011; Huettel and Kranton, 2012). For example, a neuroimaging study of the Prisoner's Dilemma has shown that mutual cooperation led to increased activation in reward regions (Rilling et al., 2002), potentially explaining how cooperative social relationships may be sustained while inhibiting the impulse to act selfishly. Many social decision-making studies have used the Ultimatum Game in which two players split a sum of money, one player proposes a division, and the other can accept or reject this. For example, it has been shown that both unfair offers and their rejection elicited activity in brain areas related to emotion, such as the anterior insular cortex, suggesting an important role for emotions in social decision-making related to cooperation (Sanfey et al., 2003). Furthermore, alpha- and theta-oscillations in prefrontal areas have been found to be sensitive to social risk and to underlie fine-tuning regulation of social decisions (Billeke et al., 2012). A study investigating whether punishment of unfair offers might be affected by the relationship between the players has shown that when the proposer was a friend rather than an unknown person, unfair offers were much less frequently rejected. The anterior prefrontal cortex plays an important role in these kind of interpersonal economic interactions (Campanhã et al., 2011).

Rodent work has also provided interesting insights into the emotional and neurobiological bases of competition. For example, water-deprived rats in a pair competing for a single source of water quickly establish a firm relationship during which one rat drinks consistently more (the dominant rat) than the other (the submissive rat). However, interestingly, when the animals are exposed to severe stress, the dominants becomes less dominant, and when their submissive cagemates are administered anxiolytics, they increase their access to resources at the expense of that obtained by dominants (Joly and Sanger, 1991). One brain area in particular seems to play a central role in the cost-benefit decision making related to competition: the anterior cingulate cortex (ACC). For example, the ACC is implicated in action selection and action outcome and effort monitoring, as well as signaling the use of social information (Rudebeck et al., 2006). Hillman and Bilkey (2012) provided rats with a choice whether to physically compete with a peer for a large food reward or not to compete and to obtain a small reward. It was found that ACC neurons electrophysiologically responded to competitive effort costs, assisting the rats in goal-directed decision making under social competitive conditions (Hillman and Bilkey, 2012).

### Observational fear learning

Decision-making can be strongly influenced by the way the social environment affects an individual's emotional state. An important example of this is social learning of fear (reviewed by Olsson and Phelps, 2007). Learning about potentially harmful stimuli and events is important in shaping adaptive behavior, which may be less risky if learned socially through observation and social communication. Experimentally, social fear learning can be assessed by subjecting an observer to another individual who is undergoing cued threatening experiences, which may elicit physiological and behavioral responses in the observer as if undergoing the threat him/herself. Fear responses acquired through conditioning and observation of a distressed model were expressed to both seen and unseen (backwardly masked) conditioned stimuli, whereas, fear responses acquired through verbal communication were expressed only to seen conditioned stimuli (Olsson and Phelps, 2004). This indicates that the route of social information transmission affects how information is perceived. Also genotype affects social fear learning. Carriers of the low activity variant of the common serotonin transporter polymorphism displayed more cued fear responses compared to high activity variant carriers when subjected to an observational fear learning paradigm in which subjects had to view a movie in which models received shocks in the presence of a conditioned stimulus (Crişan et al., 2009). Furthermore, personality has been investigated as modulator of social fear learning using a paradigm in which participants watched mock panic attacks while emotional (e.g., fear and panic) and skin conductance levels were assessed. It was found that emotional avoidance and anxiety sensitivity were positively associated with more self-reported fear and more severe panic symptoms to the challenge procedure (Kelly and Forsyth, 2009). Similarly, Hooker et al. (2008) found that trait neuroticism enhanced social fear learning. Finally, there are sex differences in observational fear conditioning using modeled “mock” panic attacks as an unconditioned stimulus and an associated neutral cue as conditioned stimulus, as women reported more distress to the conditioned stimulus (Kelly and Forsyth, 2007). Mechanistically, social fear learning shares neural features with classical conditioned fear, including the involvement of the amygdala, but also requires higher-level reflective mental state attribution, like involvement by the anterior cingulate cortex and the anterior insula (see Olsson and Phelps, 2007; Olsson et al., 2007; Olsson and Ochsner, 2008).

Next to humans, observational fear learning has been shown in a large number of species (see Olsson and Phelps, 2007) but most animal studies have been performed with rodents, showing that both visual, auditory as well as olfactory stimuli play an important role in social transfer of fear. For example, Jeon et al. (2010) demonstrated that mice observing demonstrators undergoing foot-shock stress displayed increased contextual conditioned freezing when subsequently placed in the observing chamber. This process was reduced, but not occluded, when an opaque partition was placed between the observer and demonstrator. In rodents in particular, olfactory cues play an important role, especially related to alarm pheromones. These may change autonomic activity and increase defensive and risk assessment behaviors (Kiyokawa et al., 2004, 2006) and are excreted in the rat's perianal region, especially by allogrooming, as seen during the social interaction between the demonstrator and observer rats (Knapska et al., 2010). Also, distress vocalizations affect fear learning. For example, when a conditioned stimulus was coupled to aversive 22 KHz ultrasonic vocalizations (USVs), observers displayed conditioned freezing (Chen et al., 2009) and the number of 22 KHz-USVs emitted by a fearful demonstrator was positively associated with the conditioned freezing response displayed by the observer (Wöhr and Schwarting, 2008). In line with human studies, familiarity between the observer and demonstrator results in higher observational fear learning (Chen et al., 2009; Jeon et al., 2010). Interestingly, not only fear or distress itself can be socially transmitted amongst rats and mice, also the predictive value of the conditioned stimulus itself. Bruchey and colleagues (2010) demonstrated that observer rats acquire a freezing response by observing fear-conditioned demonstrators, i.e., being exposed to the conditioned stimulus in the absence of the foot-shock. Thus, the observers responded to the conditioned stimulus as if they had experienced foot-shocks themselves. Whereas fear can be socially transmitted by social interaction between a previously stressed demonstrator and a naive conspecific, it has also been demonstrated that observation of a non-fearful demonstrator mouse inhibited subsequent recall of a context-shock association in observers (Guzmán et al., 2009). Thus, it seems that previous experience with a fear-naive demonstrator ‘buffered’ fear conditioning in observers (Panksepp and Lahvis, 2011), providing strong evidence for socio-emotional influences on the behavioral response to threat.

### Role of individual characteristics and personality differences

Although the mere presence of others may affect the decisions an individual makes, such as via facilitation and conformity, this modulating effect is strongly influenced both by the characteristics of the individual as well as that of its group mates, for instance by social status (Nicol and Pope, 1999), familiarity (see above; Swaney et al., 2001; Jeon et al., 2010), sex (see Choleris and Kavaliers, 1999; Piyapong et al., 2010) and social relationships between individuals in the group (e.g., Beauchamp, 2000; Schwab et al., 2008; Jolles et al., 2013b). Furthermore, consistently expressed behavioral differences between individuals that are otherwise similar to one another in terms of age, size and sex—also known as personality types or coping styles (Réale et al., 2007; Koolhaas et al., 2010)—may play a particular large role on individual decision-making (Webster and Ward, 2011). For example, bold compared to shy individuals have been found to be less responsive to changes in their social environment (Magnhagen and Bunnefeld, 2009) and their partner's behavior (Harcourt et al., 2009; Schuett and Dall, 2009), have a lower tendency to join and follow conspecifics (Ward et al., 2004), base their decisions less on social information (Kurvers et al., 2010) and display greater initiative in leadership (Harcourt et al., 2009). It is especially the interplay between these personality traits, individual characteristics and the relationships between individuals that affects an individual's decisions (e.g., van Oers et al., 2005; Schuett and Dall, 2009; Jolles et al., 2013b). Importantly, in this way individual characteristics and heterogeneity within groups may ultimately impact the dynamics of group decisions and behavior and affect the way in which the group as a whole functions in relation to the environment (Webster and Ward, 2011). For example, individual differences in risk-taking strongly affect social feedback between individuals (Harcourt et al., 2009), individuals may not be uniformly distributed within groups (Jolles et al., 2013a), and certain individuals may take leadership positions and thereby determine group decisions (King et al., 2009; Nagy et al., 2010). Surprisingly, few studies have considered the impact of individual characteristics and personality traits on the social modulation of decision-making. For example, although sex differences have been described in a wide range of cognitive and behavioral processes, investigations of sex differences in social learning are still largely neglected (review: Choleris and Kavaliers, 1999). Furthermore, despite the surge of interest in personality traits in animals, only in recent times have studies started to consider personality in the context of the crucial moderating effect of the social environment (review: Webster and Ward, 2011). Finally, both human and non-human studies as well as models on group behavior still seldom consider the impact of such heterogeneity on the rules underlying their coordination (but see e.g., Jolles et al., 2013a).

## Social stress and decision-making

The social environment in which humans and animals live is not devoid of psychosocial stress. Stressors may entail among others potential or actual conflicts with conspecifics either in the context of dominance-submission or in competition over (valuable) resources, the sheer performance of a task in front of conspecifics, and experiencing or witnessing aggression and violence. To assess the effects of social stressors on decision-making in the laboratory, tests are needed which produce reliable and reproducible stress-related effects. One such psychosocial test is the Trier Social Stress Test (TSST; Kirschbaum et al., 1993) and its variants (e.g., group-wise TSST; Von Dawans et al., 2011).

The TSST has been shown to be very effective in inducing stress as measured by questionnaires regarding stress, mood and anxiety as well as parameters indicative of the activation of the two main stress axes, i.e., hypothalamus-pituitary-adrenocortical axis (HPA-axis; cortisol) and the sympatho-adrenomedullary axis (SAM-axis; (nor)adrenaline) (e.g., Kirschbaum et al., 1999; Kudielka and Kirschbaum, 2005; Nater et al., 2005, 2006; Starcke et al., 2008; Nater and Rohleder, 2009; van den Bos et al., 2009; Foley and Kirschbaum, 2010; Cornelisse et al., 2011; Starcke et al., 2011; Maruyama et al., 2012; Vinkers et al., 2013). This stress effect is related to the social-evaluative and uncontrollable elements of the task (Dickerson and Kemeny, 2004): subjects have to deliver a speech as well as do a difficult arithmetic in front of a panel that judges their performance without much *a priori* knowledge of the procedure. Even anticipating delivery of the speech is already stressful.

The activation of the SAM-axis is often measured by salivary alpha-amylase, while activation of the HPA-axis is often measured by salivary cortisol (Kirschbaum et al., 1999; Kudielka and Kirschbaum, 2005; Nater et al., 2005, 2006, 2007; van Stegeren et al., 2006, 2008; Nater and Rohleder, 2009; Foley and Kirschbaum, 2010; Thoma et al., 2012). While the SAM-axis is strongly activated during the TSST and returns to baseline immediately or quickly thereafter, HPA-axis activity peaks 10–20 min after the TSST and returns to baseline about 60 min thereafter (e.g., Nater et al., 2005, 2006; Cornelisse et al., 2011; Starcke et al., 2011; Maruyama et al., 2012; Thoma et al., 2012; Vinkers et al., 2013). Cortisol levels in men are generally higher than in women, while in women the menstrual cycle and contraceptives in addition have a modulatory effect (Kirschbaum et al., 1999; Kudielka and Kirschbaum, 2005; Foley and Kirschbaum, 2010; Nielsen et al., 2013; but see Kelly et al., 2008). Thus, the TSST seems to be a useful laboratory test to delineate the effects of psychosocial stress on decision-making, when decision-making tasks are delivered after the TSST. It should be noted that the Cold Pressor Test has been used as well to delineate the effects of stress on decision-making. As at first glance the results between this test and the TSST on decision-making were not different its effects on decision-making will be included in the following paragraphs.

### Social stress: effects on decision-making paradigms

Following the TSST (as well as the Cold Pressor Test) several reward-based decision-making tasks have been shown to be affected (review; Starcke and Brand, 2012), i.e., the Iowa Gambling Task (IGT; Preston et al., 2007; van den Bos et al., 2009), the Balloon Analogue Risk Task (BART; Lighthall et al., 2009), the Game of Dice Task (Starcke et al., 2008), delay-discounting (Lempert et al., 2012) and a financial decision-making task (Porcelli and Delgado, 2009). Social stress paradigms have not been tested in animals with respect to reward-based decision-making. However, the data of other types of stress paradigms reveal similar effects as in humans: stress disrupts reward-based decision-making tasks in rats (Graham et al., 2010; Shafiei et al., 2012).

Thus far, only a few studies have been published on the effects of the TSST on social decision-making related paradigms. Social stress had no effects on moral decision-making, although in the stress group it was shown that the higher the salivary cortisol levels the more egoistic, and thus less altruistic, decisions were taken in highly emotional dilemmas (Starcke et al., 2011). Furthermore, social stress induced by the TSST increased pro-social behavior as measured by the Trust Game (reciprocal exchange) and the Dictator Game (altruism) (Takahashi et al., 2007; Von Dawans et al., 2012). Still in the latter game this effect seemed to be dependent of whether money was donated to a person or to an anonymous charity organization as Vinkers and colleagues (2013) observed that people donated less money to an organization following the TSST. Finally, altruistic punishment behavior in the Ultimatum Game was not affected immediately following the TSST (Von Dawans et al., 2012; Vinkers et al., 2013); however, it was affected when the task was administered 75 min after the TSST (Vinkers et al., 2013; see further below).

The overall impression from these studies is that differences are present in the consequences of social stress on paradigms that people play singly and those that involve interaction, even when virtually, with others. Von Dawans and colleagues (2012) suggest that this may be related to the workings of oxytocin, which would be released under stress and modulate the response in social decision-making in the direction of pro-social behavior and social support (see Taylor et al., 2000; Cousino Klein and Corwin, 2002; Heinrichs et al., 2003; Foley and Kirschbaum, 2010; Vinkers et al., 2013; see further below). The latter would lower the stress-response (Heinrichs et al., 2003; Foley and Kirschbaum, 2010). The data on the stress-related increase in pro-social behavior are in line with the observation that in primate species behaviors like reconciliation and consolation follow conflicts or social tension (e.g., Aureli et al., 1989; Koski et al., 2007; Fraser et al., 2008). These behaviors facilitate recovery from stress and counterbalance the negative consequences of social conflict on group-cohesion and may restore internal group-cohesion (Aureli et al., 1989; Fraser et al., 2008; but see Koski et al., 2007). For, maintaining internal cohesion is crucial as to maintain the benefits from group-living, which are related to increased possibilities to find and exploit food resources as well as lowering predation risk. Interestingly, oxycotin has been shown to promote in-group behavior and increase defensive aggression toward outsiders (De Dreu et al., 2010). To what extent this relates to the observation that altruism in the Dictator Game was enhanced following social stress depending upon whether it was in the context of persons or a charity organization (Takahashi et al., 2007; Von Dawans et al., 2012; Vinkers et al., 2013) remains to be studied. These data thus provide a link between causal mechanisms and functional mechanisms of pro-social behavior following social stress. The biological meaning of the data on reward-based decision-making is discussed in section Timing, coping styles and daily life (coping-styles).

### Social stress: sex differences

Studies directed at dissecting sex differences showed that men displayed more risk-taking behavior following stress (IGT and BART), whilst women were more risk-aversive (BART) or became more task-focused (IGT). These studies also showed that sex differences were related to the levels of cortisol. The higher the levels of cortisol, the more risk-taking behavior was shown by men (IGT; van den Bos et al., 2009). Women, on the other hand displayed more risk-aversive or task-focused behavior with increasing levels of cortisol (BART; Lighthall et al., 2009; IGT; van den Bos et al., 2009). Data from the IGT also indicated that women became more risk-taking when levels were too high (van den Bos et al., 2009; see also Witbracht et al., 2012). Thus, overall these data indicate that stress has a different effect on reward-based decision-making in men and women with different underlying effects of cortisol. This was recently confirmed using the Cambridge Gambling Task and a job assessment procedure to induce stress: while salivary cortisol levels were positively correlated with risk-taking behavior in men, they were if anything weakly negatively correlated in women (van den Bos et al., 2013b). Interestingly, this study also revealed a different relationship between salivary alpha-amylase and risk-taking in men and women: while in women a positive relationship was found, a negative relationship existed in men (van den Bos et al., 2013b). These data underline that differences do exist between men and women regarding the relationship between stress, neuro-endocrine changes and decision-making (see also de Visser et al., 2010; van den Bos et al., 2013a).

Studies on social decision-making have been mainly done in male only populations (Takahashi et al., 2007; Von Dawans et al., 2012; Vinkers et al., 2013) or do not mention potential sex-differences in the data set (Starcke et al., 2011), precluding therefore to discuss differences between men and women in this respect. Still, one study using the same social-decision-making tasks and stress protocol as applied in men, did not observe an effect of social stress on social-decision making in women (Koot, unpublished). None of the studies in men reported a relation with cortisol (Von Dawans et al., 2012; Vinkers et al., 2013). Furthermore, while one study reported a correlation between heart-rate and pro-social behavior (Von Dawans et al., 2012), other studies did not observe a correlation between salivary alpha-amylase and pro-social behavior (Takahashi et al., 2007; Vinkers et al., 2013).

The increase in risk-taking behavior in men in reward-related decision-making may be associated with a loss of top-down control of prefrontal over subcortical areas, such as mediated by the lateral orbitofrontal cortex and dorsolateral prefrontal cortex (Piazza and Le Moal, 1997; Arnsten, 1998, 2009; Erickson et al., 2003; Stark et al., 2006; Wang et al., 2007; Kern et al., 2008; Dias-Ferreira et al., 2009; Goldstein et al., 2010; Koot et al., 2011, 2013). Furthermore within the limbic system high levels of cortisol may shift the balance of the activity of the ventral striatum (reward-related behavior) and amygdala (punishment-related behavior) toward the ventral striatum (Piazza et al., 1993; Dellu et al., 1996; Piazza and Le Moal, 1997; Pruessner et al., 2004; Mather et al., 2010; Porcelli et al., 2012; see Wager et al., 2008). A recent study showed that increasing noradrenergic activity decreased amygdala activity and processing of fearful faces (Schwabe et al., 2013). Thus, it may be hypothesized that in men the prefrontal-subcortical balance is disrupted by acute stress. In line with this, it was recently observed that systemic injections of corticosterone in male rats in a rodent analogue of the Iowa Gambling Task (de Visser et al., 2011) disrupted decision-making performance, which was associated with changes in activity in prefrontal structures (Koot et al., 2011, 2013). Still it should be noted that such effects of corticosterone were not observed in other studies (Graham et al., 2010; Shafiei et al., 2012). However, as discussed by Koot et al. (2013) this may be due to the way these studies applied corticosterone and/or administered the task following corticosterone injections. In general, studies on stress in male rats have revealed that acute stress sensitizes the reward system (through corticosterone; e.g., Piazza and Le Moal, 1997). As mentioned above it has been suggested that stress induced release of oxytocin may have an effect on the way subjects engage into social decision-tasks. Currently no studies exist which have studied the interaction between stress, changes in neural structures and social decision-making tasks.

As to the underlying neural substrate in women it seems that top-down control may actually be increased under stress, related to levels of cortisol, with among others a lower striatal and a stronger amygdala activity (Stark et al., 2006; Wang et al., 2007; Mather et al., 2010; Porcelli et al., 2012). A recent study showed that increasing noradrenergic activity increased amygdala activity, decreased orbitofrontal activity (thereby decreasing top-down control) and increased processing of fearful faces (Schwabe et al., 2013). It has been suggested that the persistent activity in for instance the anterior cingulate cortex following a stressful experience in women may be associated with the development of depressive symptoms in women related to tendencies of ruminative thinking (Tamres et al., 2002; Wang et al., 2007). The menstrual cycle has a strong effect on the outcome of changes in neuronal activity (Goldstein et al., 2010; Ter Horst et al., 2013). Thus, at present changes in neural activity in women are less clear and straightforward than in men. However, by and large these changes in women seem compatible with a shift toward risk-aversive behavior. Like in men, currently studies in women are lacking which have looked at the interaction between stress, changes in brain structures and social decision-making tasks.

### Timing, coping styles and daily life

For a full understanding of the effects of social stress on decision-making paradigms three issues need further discussion: (1) short-term *versus* long-term effects of stress (timing), (2) relationship between stress, coping styles and task performance, and (3) consequences for daily life.

While most studies have applied decision-making tasks directly following the TSST, the data of several studies suggest that stress, notably cortisol, may have time-dependent effects on the balance between prefrontal and subcortical functioning. These timing effects may be related to non-genomic, rapid, and delayed, genomic, corticosteroid actions. For instance, when targeting these two time-domains specifically by administering cortisol in human subjects either shortly or several hours before behavioral testing, working memory was found to be improved by slow compared to rapid corticosteroid actions, and this improved performance was linked to enhanced activity in the dorsolateral prefrontal cortex (Henckens et al., 2011). These and other studies have led to the hypothesis that prefrontal cortical functioning is impaired by corticosteroids acting via rapid non-genomic pathways, but enhanced by slow corticosteroid actions (Joëls et al., 2012). Few studies have targeted these different time-windows thus far. In a recent study using the TSST and a social decision-making task it was shown that male subjects showed more acceptance of ambiguous offers when the task was administered 75 min after the TSST than when administered immediately thereafter, leading the authors to conclude that this may be due to enhanced cognitive control, although it should be mentioned that no direct relationship with cortisol levels was found (Vinkers et al., 2013). Accordingly, it may be suggested that the effect of psychosocial stress on decision-making may be different when tasks are administered immediately following a stressor or sometime thereafter. It is clear that this needs further study.

In men, it seems that high levels of cortisol following a stressor are related to risk-taking: high-cortisol responders show decreased IGT performance, while non/low-cortisol responders do not (van den Bos et al., 2009). However, these data seem to be in contrast with data on coping styles. Male subjects with a pro-active coping style are in general considered to be more risk-taking than male subjects with a reactive coping style (Koolhaas et al., 1999, 2010; Coppens et al., 2010). Subjects with a reactive coping style show a higher HPA-axis activity than subjects with a pro-active coping style, while subjects with a pro-active coping style show a higher SAM-axis activity than subjects with a reactive coping style (Koolhaas et al., 1999, 2010; Coppens et al., 2010). This apparent contradiction may be resolved when the stress response and the task are considered separately. Thus, the stress response in the TSST is directed to the challenge, i.e., the speech and arithmetical task in front of the panel. The physiological and neural changes would normally allow the organism to cope with this particular challenge. In case of the non/low-cortisol responders, which have a short-lasting SAM-axis activation during the TSST, this would be to take immediate action directed toward this particular stressor with an already learned response or routine (Koolhaas et al., 1999, 2010; Coppens et al., 2010). As the SAM-axis is strongly activated during the task with little effect thereafter, there is no after-effect when the IGT is administered. In case of the cortisol responders, which also have a short-lasting SAM-axis activation during the TSST and a long-lasting HPA-axis activation, the coping response is to “freeze” i.e., re-assess the situation by exploration and being sensitive to environmental cues (Koolhaas et al., 1999, 2010; Coppens et al., 2010). Given the observed effects of stress and cortisol on neural structures, their brain seems to be in a “exploratory mode,” i.e., a decreased prefrontal activity and an increased ventral striatal (dopaminergic) activity in which risk-taking, as part of exploratory behavior, is included (Fiorillo et al., 2003). This “exploratory mode” seems to be set in motion during the TSST and remains for some time thereafter. This “exploratory mode” may be followed by an enhanced level of cognitive control (Vinkers et al., 2013), likely to be mediated by increased activity in the prefrontal cortex (Henckens et al., 2011), which may serve to store newly obtained information and/or regain homeostasis and cognitive control. Thus, when the IGT is administered shortly after the TSST, the on-going coping response interferes with IGT task-performance. For, the IGT or other decision-making tasks require a delicate balance between cognitive control and reward/punishment-sensitivity or a prefrontal-subcortical balance (de Visser et al., 2011). As the brain of cortisol-responders is in a “exploratory/risk-taking mode” they are more sensitive to the immediately highly rewarding decks of cards, which is indeed the case as judged from their choice behavior (van den Bos et al., 2009). One may speculate that a different pattern emerges when the decision-making task in itself would be stressful. In this case, subjects with a pro-active coping style would probably take more risks than subjects with a reactive coping style as in this case the coping style is directly related to the task. It is clear that this needs further study. Data on rat behavior in resident-intruder paradigms in which pro-active copers show a fight-flight response and reactive copers a freezing response suggest such differences in risk-taking tendencies during interactions (Koolhaas et al., 1999, 2010).

Discussions on coping styles have nearly exclusively focused on differences in behavior in male subjects (Koolhaas et al., 1999, 2010; Coppens et al., 2010). While it has been suggested that male and female subjects in general differ in coping style, for instance fight-flight (pro-active) *versus* tend-and-befriend (reactive; Cousino Klein and Corwin, 2002) or problem-oriented (pro-active) *versus* emotion-oriented (reactive; see Tamres et al., 2002 for discussion), this is too limited a view as the differences in coping styles in male subjects already show. The current data rather suggest that a distinction in female subjects may also occur in coping styles, with in all likelihood “tend and befriend” as the most dominant one (Taylor et al., 2000; Cousino Klein and Corwin, 2002; Tamres et al., 2002) and likely to be a reactive coping style (Koolhaas et al., 2010). This coping style is related to the workings of oxytocin (Cousino Klein and Corwin, 2002), directed at caring for the young and/or seeking social support (Taylor et al., 2000; Tamres et al., 2002) and, possibly, leading to a reduction of HPA-axis activity (Cousino Klein and Corwin, 2002). Thus, the cortisol-mediated increase in risk-aversive behavior in reward-based tasks may be related to changes in neural structures toward safety and social support. Yet, it is clear that further studies are needed to substantiate this.

As risk-taking is in general considered to be a more disastrous mode of behavior than risk-aversiveness, the behavior of women following psychosocial stress may be considered as less detrimental than the behavior of men. However, this may not be entirely true. For instance, trying to rescue someone from a burning house with an objectively high risk of death or injury in the course of action is as disastrous as not-rescuing someone from a burning house with an objectively low risk of death or injury in the course of action. So it may be rather the context that gives one behavioral pattern an advantage over another or not than the attitude *per se*: today's hero may be tomorrow's fool and *vice versa*.

## Laboratory studies and real-life studies

The laboratory environment offers the growing field of social neuroscience the opportunity to study general principles underlying brain-behavior relationships in a social context by using well-defined tasks tailored to be performed in and outside of scanners (review; Rilling and Sanfey, 2011). Similarly, the laboratory is well suited to study general effects of the social environment on individual behavior as for instance indicated in the previous section and as shown for instance by the effects of peers on risk-taking behavior (Gardner and Steinberg, 2005). However, at the same time it is often difficult, if not impossible, in laboratory settings to arrange the complete array of social contexts and/or social interactions. This becomes especially problematic when the behavior of the individual in relation to the social environment is the focus of attention, especially such as related to understanding the development of behavioral pathologies, intervention strategies and monitoring the success of therapies. For instance, in the case of the development of social conduct disorders probably not all social settings or social partners are equally likely to trigger a response, i.e., they may strongly differ between subjects and even across the life-span of subjects. As argued by others, *time*, i.e., when events relevant to the individual occur, and *context*, i.e., where and with whom events occur, are the limiting factors in laboratory studies (Johnson et al., 2009). Therefore, it would be ideal to study social interactions and their effect on the individual in real life as a complement to laboratory studies.

Among the most promising approaches to understanding time-limited behaviors in ecologically valid circumstances involves ambulatory monitoring through mobile technologies. This strategy is known alternately as Ecological Momentary Assessment (EMA) as well as the Experience Sampling Method (ESM), and it uses devices such as smart phones or other portable microcomputers to collect data at numerous intervals throughout the day. Like all methods, EMA/ESM also has its own limitations that include the necessity for all repeated assessments to remain brief as well as its reliance on subjective reports from the individual. However, extensive validation studies in diverse normal and psychiatric populations have demonstrated the feasibility and validity of this technique as a means of assessing psychological states and behavior in real time and in natural contexts (Granholm et al., 2008; Johnson et al., 2009; Husky et al., 2010). The major contribution of EMA/ESM is that it should provide a bridge between laboratory-based protocols with daily life behaviors that are otherwise inaccessible to the researcher. The value of EMA/ESM in investigating dynamic daily life risk factors has already been demonstrated relative to substance use behavior (Swendsen et al., 2000, 2011; Epstein et al., 2009), depressive cognitions (Swendsen, 1998), and many other “micro” processes of daily life.

Studying the dynamic character of social interactions including its long-term effects on the individual is standard in studies involving primates, either in the wild or in the laboratory. Still, it is clear that opportunities and possibilities for invasive neuroscience research are limited. In rodent studies, however, the opposite pattern seems to be present: while many experiments are directed at understanding the neural basis of for instance decision-making behavior in stand-alone tasks (de Visser et al., 2011), in general relatively little research is done in a long-term social context, an exception being for instance the work by Blanchard and Blanchard (1989), Blanchard et al. (1995, 2001, 2002). Recently home-cage tests have been developed allowing to address decision-making in a more naturalistic setting, i.e., to combine cognitive testing with a social/ecological-like environment (e.g., *Automated group-cage* (AGC), PhenoSys GmbH, Berlin, Germany; *Intellicage* (IC), Newbehavior AG, Zurich, Switzerland). In these home-cages, mice or rats are free to move and interact with each other but they can also voluntarily access operant modules situated inside or outside the home-cage. Using the *Home Cage Panels* (PRS Italia, Rome, Italy) Zoratto and colleagues (2013) developed a setting where animals were pair-housed, but could be tested singly. Furthermore, to assess social behavior in more detail programmes have been developed to analyse social interactions (De Chaumont et al., 2012). Thus, future studies in social neuroscience in rodents should develop protocols that combine new possibilities of studying the role of neural structures in behavior, such as by optogenetics, with well-defined and controlled home-cage social settings. This would allow inducing changes in behavior due to changes in neural structures within a social context in a controlled way.

## Concluding remarks

A large extent of the exceptional success of the human species is related to our complex social life. It is therefore important to properly understand in what way the social environment may modulate decision-making. In the foregoing sections we have highlighted research on this topic, both from human and animal-based neuropsychological studies as well as insights from a behavioral ecology perspective. It is the combination of these top-down and bottom-up approaches that may enable us to fully understand decision-making and the social factors that affect it.

Although humans are outperforming animals regarding social skills, rodents and other animals bear some fundamental aspects of these skills as well, indicating the important role of social influences on decision-making in evolution. These comparisons may therefore not only enable us to better understand our own behavior, they may help us understand the ways in which our behavior may be fundamentally similar or different from that of other animals. However, with a few exceptions—like the observational fear learning task and Prisoner's Dilemma game—these kinds of translations between human and rodent based neuropsychological studies and behavioral ecology studies have rarely been done.

Future studies on social modulation of decision-making can therefore benefit by making the links between both fields of research and taking both a top-down and bottom-up approach. Furthermore, this may enable us to go beyond general social modulating effects and allow to understand how individual characteristics and heterogeneity within groups affect decisions that individuals make and the way this may ultimately affect group functioning, such as can be seen in human society. For instance, future experiments should focus on further dissecting the interaction between social stress, gender and decision-making taking timing, the kind of decision-making task and coping style into account. While in rats social stress has been mainly studied in the context of coping styles and neuro-endocrine changes (Koolhaas et al., 1999, 2010) as well as long-term consequences related to depression and buffering effects of the social environment hereon (e.g., Blanchard et al., 1995, 2001, 2002; Von Frijtag et al., 2000), no studies exist which have explicitly looked at decision-making either related to food, social partners or otherwise. Clearly such studies are needed in parallel to human studies to unravel short- and long-term effects of social stress in male and female subjects.

As argued, studies are needed in humans and animals under real-life conditions to assess the impact of (stressful) events on subsequent decisions. For instance, Newman and colleagues (2007) showed that daily hassles affected the decision to eat in female subjects in real life. Such studies clearly help in understanding both similarities and discrepancies between findings in real life and laboratory findings and thus enhance the application of laboratory-acquired knowledge in real-life conditions.

For a successful cross-species approach it is mandatory to show that the same principles underlie changes in decision-making behavior regarding social interactions and stress. This is all the more important when focusing e.g., on sex differences (van den Bos et al., 2013a). Thus, this requires understanding social behavior and its underlying principles in the context of behavioral ecology as outlined under section Decision-making in a social context for instance. Furthermore, it requires to use paradigms which in a species-specific way tap-off similar phenomena and/or are matched as closely as possible (see de Visser et al., 2011; van den Bos et al., 2012, 2013a; Jimura et al., 2013).

As a final remark, we do hope that this review may serve as a fruitful starting point for extending current studies and discussions of decision-making by incorporating the social environment.

### Conflict of interest statement

The authors declare that the research was conducted in the absence of any commercial or financial relationships that could be construed as a potential conflict of interest.
